# Why the short face? Developmental disintegration of the neurocranium drives convergent evolution in neotropical electric fishes

**DOI:** 10.1002/ece3.2704

**Published:** 2017-02-15

**Authors:** Kory M. Evans, Brandon Waltz, Victor Tagliacollo, Prosanta Chakrabarty, James S. Albert

**Affiliations:** ^1^ Department of BiologyUniversity of Louisiana at LafayetteLafayetteLAUSA; ^2^ Universidade Federal do TocantinsPrograma de Pós‐graduação Ciências do Ambiente (CIAMB)PalmasTocantins77001–090Brazil; ^3^ Department of BiologyLouisiana State UniversityBaton RougeLAUSA

**Keywords:** developmental bias, geometric morphometrics, homoplasy, integration, modularity

## Abstract

Convergent evolution is widely viewed as strong evidence for the influence of natural selection on the origin of phenotypic design. However, the emerging evo‐devo synthesis has highlighted other processes that may bias and direct phenotypic evolution in the presence of environmental and genetic variation. Developmental biases on the production of phenotypic variation may channel the evolution of convergent forms by limiting the range of phenotypes produced during ontogeny. Here, we study the evolution and convergence of brachycephalic and dolichocephalic skull shapes among 133 species of Neotropical electric fishes (Gymnotiformes: Teleostei) and identify potential developmental biases on phenotypic evolution. We plot the ontogenetic trajectories of neurocranial phenotypes in 17 species and document developmental modularity between the face and braincase regions of the skull. We recover a significant relationship between developmental covariation and relative skull length and a significant relationship between developmental covariation and ontogenetic disparity. We demonstrate that modularity and integration bias the production of phenotypes along the brachycephalic and dolichocephalic skull axis and contribute to multiple, independent evolutionary transformations to highly brachycephalic and dolichocephalic skull morphologies.

## 1 Introduction

Convergent evolution is the independent phylogenetic origin of a similar form or function in different taxa and is often viewed as strong evidence for the influence of natural selection on molding organismal phenotypes (Futuyma, 1998; Gallant et al., 2014). The concept of convergence helped shape the understanding of adaptation and the role of adaptive radiation in the framework of evolutionary biology (Losos & Miles, 2002; Schluter, 2000). Convergent evolution has been reported in numerous clades (Adams & Nistri, 2010; Mahler, Ingram, Revell, & Losos, 2013; Rüber & Adams, 2001; Wroe & Milne, 2007). In its most functional understanding, convergence is viewed as evidence of similar environmental demands independently producing similar phenotypes designed to meet those demands.

In the neo‐Darwinian paradigm, phenotypic variation was deliberately modeled as continuous and isotropic (i.e., unbiased) with respect to the adaptive need of the organism (Charlesworth, Lande, & Slatkin, 1982; Dobzhansky, 1970). This view of variation has strong predictive power in many comparative genetic studies of wild and laboratory populations, in part due to the additive nature of genetic variation in which phenotypic variance arises from the average effects of many alleles, each with small effects on the phenotype (Futuyma, 2015). In the neo‐Darwinian paradigm, the external environment is treated as the principle source of information affecting phenotypic evolution and organisms are largely regarded as passive objects with little or no capacity to influence the nature or direction of their evolutionary trajectories, see discussions in (Arthur, 2004; Wagner & Altenberg, 1996). The view of unbiased variation was advanced by the architects of the synthesis to expunge vague notions of vitalism and orthogenesis that had plagued earlier generations of researchers (Mayr, 1982).

The emerging synthesis of evolutionary and developmental biology (evo‐devo) reflects an alternative view of organisms as more active agents in the evolutionary process (Hall, 1999; Raff, 1996; Simpson, 1953; Wagner & Zhang, 2011; West‐Eberhard, 2003). The evo‐devo approach recognizes how biases in the production of variation can channel the formation of novel phenotypes (Watson, Wagner, Pavlicev, Weinreich, & Mills, 2014), constrain the tempo and mode of evolution (Wagner & Zhang, 2011) and have predictable effects on evolutionary trends (Stern, 2000; Yampolsky & Stoltzfus, 2001).

Developmental biases on the production of phenotypic variation may channel the evolution of convergent forms by limiting the range of phenotypes produced during ontogeny (Smith et al., 1985). Classic examples of developmental biases include patterns of body segmentation via conserved *Hox* gene expression patterns, limb loss in tetrapods and patterns of digit loss in amphibians (Lande, 1978; Wake, 1991; Wake, Wake, & Specht, 2011). By biasing the direction of phenotypic variation in development, some phenotypes can be produced at higher frequency than others. These asymmetries can result in seemingly convergent phenotypes by chance (stochastically) without the need for natural selection from the environment, although natural selection may still filter out produced phenotypes (Smith et al., 1985).

The study of modularity is an emerging field within evo‐devo that assesses the covariation among traits in the presence of genetic and environmental variation. Phenotypic modules are quasi‐independent anatomical parts of organisms, which are tightly integrated internally in terms of embryological, physiological, or functional characteristics, but which may evolve independently among lineages relative to other modules (Schlosser & Wagner, 2004; Wagner & Altenberg, 1996). The degree of covariation among traits in development can have strong implications on the production of phenotypic variation and patterns of adaptive diversification (Gould, 1966; Kirschner & Gerhart, 2006; Schlosser & Wagner, 2004; Wagner & Altenberg, 1996). Whereas developmental modularity facilitates functional specialization and differentiation of body parts, developmental integration may coordinate patterns of variation among correlated traits as a result of a complex underlying pleiotropic network (Draghi & Wagner, 2008; Marroig, Shirai, Porto, de Oliveira, & De Conto, 2009). The evolution of integration has been hypothesized to constrain the range of phenotypic evolution, as a complex underlying pleiotropic network would globalize the effects of genetic changes between both modules creating an inertial force thus limiting the capacity for an integrated system to respond to selection (Marroig et al., 2009). The degree of covariation can therefore affect rates of phenotypic evolution and functional specialization (Hallgrímsson et al., 2009). The degree of covariation among traits may also evolve, thereby changing the evolvability of a structure by increasing its capacity to respond to selection (Draghi & Wagner, 2008; Wagner & Altenberg, 1996; Wagner & Zhang, 2011).

The skull was a key innovation in the evolution of vertebrates and is a popular model for the study of modularity. The skull has been re‐adapted in almost every major vertebrate lineage and performs a wide range of functions, including protecting the brain and special sense organs, and as structural support and muscle attachment sites for tissues involved in respiration, feeding, and communication behaviors of the oral jaws and pharynx (Barbeito‐Andrés, Gonzalez, & Hallgrímsson, 2016; Hanken & Hall, 1993a, 1993b). Within the skull, two developmentally distinct modules have been identified: the face and braincase (Marroig et al., 2009; Piras et al., 2014; Porto, Shirai, Oliveira, & Marroig, 2013; Sanger, Mahler, Abzhanov, & Losos, 2012; Tokita, Kiyoshi, & Armstrong, 2007). Despite being partially distinct developmental modules, the face and braincase are largely considered to be integrated in development and evolution (Álvarez, Perez, & Verzi, 2015; Collar, Wainwright, Alfaro, Revell, & Mehta, 2014; Klingenberg & Marugán‐Lobón, 2013; Kulemeyer, Asbahr, Gunz, Frahnert, & Bairlein, 2009; Piras et al., 2014).

Within the skull, a potential developmental bias may lie in patterns of craniofacial ontogeny. Variation in facial development has been linked to changes in the signaling from the Frontonasal ectodermal zone (FEZ), a developmental field located anterior to the forebrain and juxtaposed between the *Fgf8* and *Shh* signaling centers (Hu & Marcucio, 2009a; Hu, Marcucio, & Helms, 2003; Hu et al., 2015; Whitehead & Crawford, 2006; Young et al., 2014). In a developmental study of amniotes, disruptions in *Fgf8* and *Shh* signaling from the forebrain resulted in failure of the FEZ to induce expansion of the face (Hu & Marcucio, 2009a, 2009b; Hu et al., 2003, 2015; Marcucio, Cordero, Hu, & Helms, 2005). As a result, embryos were born with truncated faces; however, the nasal capsule and structures located just posterior to the nasal capsule were well formed. Thus, *Fgf8* and *Shh* signaling from the forebrain may be an integrating factor that spans both the braincase and facial modules. Brachycephalic species with foreshortened skulls may have evolved by reduced efficacy of these signaling molecules from the forebrain region. A direct genetic basis for the disruption of *Fgf8* and *Shh* signaling from the forebrain is difficult to ascertain due to the highly complex pleiotropic nature of the genotype–phenotype map (Wagner & Zhang, 2011). It is likely that the disruption of these signaling molecules from the forebrain is a plastic response to a mutation of one or more genes within the large network of genetic interactions that govern skull development. This pleiotropic network is also expected to have a large mutational target size, such that a mutation in any of several candidate genes would result in a similar truncated response (Boell, 2013; Houle, 1998). This plastic response would bias the phenotypic variation toward the production of brachycephalic skulls. This bias could therefore result in multiple independent evolutionary transformations of brachycephalic skull shapes. Large pleiotropic networks governing skull development have been noted in both mammals and fishes (Cooper, Wernle, Mann, & Albertson, 2011; Martínez‐Abadías et al., 2012).

Here, we study the interface between developmental modularity and integration and the consequences of each on patterns of neurocranial shape diversity and variation using two‐dimensional geometric morphometrics. We assess variation in the relative skull length, during ontogeny and through phylogeny, in gymnotiform electric fishes, a diverse clade of tropical fishes from Central and South America. Gymnotiforms are notable for their high diversity of craniofacial phenotypes, including extremely brachycephalic and dolichocephalic taxa, and many species with intermediate skull phenotypes (Albert, 2003; Carvalho & Albert, 2015; Ivanyisky & Albert, 2014). These phenotypes have evolved multiple times within Gymnotiformes and have led several investigators to hypothesize different selective forces that could be driving their recurrence (Hilton, Fernandes, & Armbruster, 2006; Marrero & Winemiller, 1993). We test for the effects of developmental integration and modularity on ontogenetic disparity and adult relative skull length. We also test for possible biases in the production of brachycephalic over dolichocephalic skull shapes by quantifying the extent of convergent evolution along this trait axis. We hypothesize that developmental modularity produces brachycephalic skulls and that developmental integration produces dolichocephalic skulls as a result of the integrating effect of signaling molecule patterns that traverse both face and braincase regions during development. We further hypothesize that this truncation reduces total neurocranial ontogenetic disparity in such a way that modular species exhibit less ontogenetic disparity while integrated species exhibit more ontogenetic disparity. Finally, we hypothesize that developmental biases may have contributed to widespread homoplasy in relative skull length among extant gymnotiform species (Sadleir & Makovicky, 2008; Sanger et al., 2012; Wroe & Milne, 2007).

## 2 Methods

### 2.1 Study system

Gymnotiform electric fishes are known from 220 species representing five families and 35 genera. Gymnotiformes occupy a wide range of aquatic habitats in the lowland Neotropics, from deep (to 85 m) channels, and floodplains of large lowland rivers to rapids in the mountain streams of the Brazilian shield and Andean piedmont above 1,000‐meter elevation (Carvalho, 2013; Crampton, 2011). Within Gymnotiformes, much of the phenotypic disparity is restricted to the craniofacial region making this clade an excellent system for which to study the evolution of craniofacial diversity. Skull and snout shapes in Gymnotiformes range from the foreshortened bulldog‐shaped faces of the hypopomid *Brachyhypopomus* and the apteronotids *Adontosternarchus* and *Sternarchella*, to the elongate tubular snouts of the rhamphichthyid *Rhamphichthys* and the apteronotids *Orthosternarchus* and *Sternarchorhynchus,* with other gymnotiform taxa exhibiting a range of intermediate skull and snout phenotypes. These specialized head and snout morphologies have been hypothesized to represent convergent adaptations for the utilization of trophic resources (Albert, 2001; Albert & Crampton, 2009; Ellis, 1913; Marrero & Winemiller, 1993; Winemiller & Adite, 1997).

### 2.2 Specimen selection and preparation

Specimens used in this study were collected from multiple field localities throughout northern South America (particularly the Western Amazon Basin) under collecting permits from national authorities and deposited in museum collections. Specimens were collected by trawling deep river channels or dip‐netting in small streams, depending on species’ habitat preferences.

Specimens were cleared and stained for bone and cartilage following the method of Taylor and Van Dyke, (1985), with the addition of xylene washes to remove excess lipids (Ivanyisky & Albert, 2014). Adult neurocrania were selected for geometric morphometric analyses based on degree of ossification of endochondral bones in the sphenoid region (Albert, 2001), and by the inflection point in the growth curve of relative head length (Hulen, Crampton, & Albert, 2005). Neurocrania examined for osteology were dissected under an Olympus SZX‐12 stereomicroscope, and photographed in lateral views using a Nikon Coolpix digital camera with specimen orientations standardized to limit the effects of rotation and orientation. Specimens too large to be cleared and stained were radiographed using a Kevex MicroFocus X‐ray source at the Academy of Natural Sciences in Philadelphia, or using a Varian PaxScan image receptor in a Faxitron cabinet set at 33 kV at Louisiana State University. Damaged or deformed specimens were excluded. Digital images were imported and converted into *tps* files using the *tpsUtil* program.

### 2.3 Geometric morphometrics

**Figure 1:**
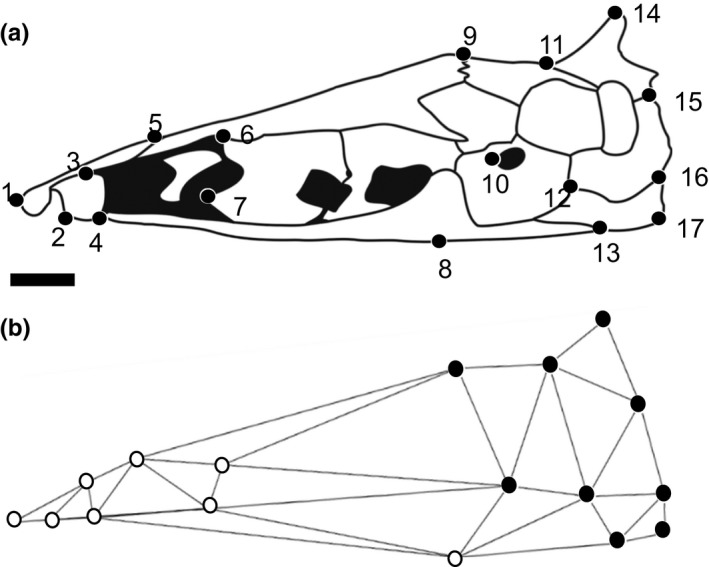
Line drawings of the neurocranium of *Sternarchella schotti* in lateral view. (a) Landmarks (*n* = 17) used in geometric morphometric analyses of gymnotiform fishes. (b) Wireframe drawing of neurocranium in panel (a), with landmarks categorized to face (in white dots) and braincase (in black dots) developmental modules. Anterior to left. Scale bar = 1.0 mm

Two‐dimensional geometric morphometrics was used to capture changes in the shape of neurocranial morphology in lateral view (Adams, Rohlf, & Slice, 2004; Mitteroecker, Gunz, & Bookstein, 2005; Thompson, 1942). Images were digitized in *tpsDIG2* (Rohlf, 2006) by placing digital markers on homologous landmarks selected to cover as much of the image as possible (Figure 1a; Table 1). Digitized files were imported into *MorphoJ* and a full Procrustes fit was used to translate the landmarks into a common coordinate space. This superimposition scales the specimens to unit centroid size and rotates them relative to each other so as to minimize the distances between homologous landmarks on different specimens (Ruber & Adams, 2001). By doing this, the effect of shape and size was separated and the variation in the position and orientation of specimens was removed (Klingenberg, Barluenga, & Meyer, 2003).

**Table 1:** Definitions of the 17 landmarks (LM) of the neurocranium in lateral view used in the geometric morphometric analysis of Gymnotiformes

LM#	Definition
1	Most anterior point of Mesthmoid
2	Most anterior point of Ventral Ethmoid
3	Posterior margin of Ventral Ethmoid and Mesethmoid
4	Parasphenoid/Ventral Ethmoid suture
5	Frontal/Mesethmoid suture
6	Anterior Frontal/Orbitosphenoid suture
7	Most anterior lower projection of Orbitosphenoid
8	Lower ridge of Parasphenoid
9	Frontal/Parietal suture
10	Most anterior point of Prootic Foramen
11	Supraoccipital/Parietal suture
12	Basioccipital/Exoccipital/Prootic intersection
13	Parasphenoid/Basioccipital suture
14	Most superior inflection of Supraoccipital
15	Supraoccipital/Exocciptial suture
16	Exoccipital/Basioccipital suture
17	Posterior corner of Basioccipital

For the macroevolutionary analysis of skull evolution, a total of 157 morphologically mature specimens representing 133 species and all 35 recognized genera were analyzed for the study of neurocranial evolution (Table S1). Gymnotiformes represent a typical condition in Neotropical fishes where much of their diversity is distributed in allopatry is difficult to reach places and difficult to collect due to remote localities and sociopolitical unrest in many of the regions. Furthermore, many species are rare in collections and in many cases only known from one or a handful of specimens. As a result, many of our species are represented by a single adult specimen. This could present difficulties in the interpretation of our data as many factors can influence skull shape in this clade (i.e., ontogeny and sexual dimorphism). To standardize for these factors, only mature adult specimens were sampled for each species and in cases where species were reported to exhibit sexual dimorphism of the snout and jaws (a common condition in Apteronotidae), only adult males were sampled in an effort to capture the maximum disparity of each species in our analysis.

Shape changes in neurocrania associated with growth were assessed for 17 gymnotiform species and all recognized gymnotiform families, from a total of 363 individual specimens representing an average of 21.4 specimens per species (Table S2). We use size series of different individuals sampled from the same population as a proxy for ontogenetic growth. Fishes and other poikilothermic vertebrates exhibit indeterminate growth, in which body size is a better measure of ontogenetic age than is clock or calendar time (Kirkpatrick, 1984), and this has been demonstrated in a laboratory‐raised species of gymnotiform electric fish *Apteronotus leptorhynchus* (Ilieş, Sîrbulescu, & Zupanc, 2014). To date, only five gymnotiform species have been raised in captivity, all of which are species adapted to small streams, and no riverine species has yet been raised in captivity (Kirschbaum & Schwassmann, 2008). Therefore, as with the great majority of fish species, most information on gymnotiform ontogeny has been documented by comparing wild‐caught specimens of different sizes (Albert & Crampton, 2009; Hilton et al., 2006).

Specimens selected for the ontogenetic size series were limited to individuals collected at the same time and place to reduce the potential effects of environmental variation. Most of the species are represented by specimens collected from a single trawl pull, thereby representing members of a single breeding population. This collecting and sampling filter removes much of the phenotypic variation associated with geographic and habitat variation. Specimens were also selected to represent as large a range of body sizes as possible from among available materials. This approach does however confound static and ontogenetic allometry as it captures all shape variation associated with size and not just the shape variation associated with growth (Pélabon et al., 2014; Voje & Hansen, 2013; Voje, Hansen, Egset, Bolstad, & Pelabon, 2014). For most species, these specimens range in size from posthatching juveniles just at the onset of bone mineralization, to morphologically mature adults >90% maximum known total length.

### 2.4 Principal components analyses

A principal components analysis (PCA) was conducted from a covariance matrix of Procrustes coordinates and used to analyze the differences and similarities in shape among specimens. This analysis displays the PC scores as scatter plots and yields new variables for other types of statistical analyses. PCA results were displayed using a ball‐and‐stick model generated by *MorphoJ*, showing the transposition of individual landmarks on the X‐ and Y‐axes, using the mean distribution between the landmarks as a starting point, and drawing a line of best fit between the remaining landmark positions (Klingenberg, 2011). The ball‐and‐stick model was also superimposed on a deformation grid generated by the thin‐plate spline method. A thin‐plate spline is an interpolation technique that shows the movement of residuals on the x‐ and *y*‐axis in a two‐dimensional grid plane (Zelditch, Swiderski, & Sheets, 2012). Changes in the location of the residuals can bend and contort the frame to show the differences in the relative positions of homologous landmarks.

Scores from the first principal axis of our analysis are used in several of our subsequent analyses to study the evolution of a specific aspect of shape change (relative skull length). Several cautions against the explicit use of PC1 in macroevolutionary analyses are voiced in (Bookstein, 2015; Mitteroecker, Gunz, Bernhard, Schaefer, & Bookstein, 2004; Mitteroecker et al., 2005; Uyeda, Caetano, & Pennell, 2015). However, to date, the use of principal components analysis remains a popular way to model individual aspects of shape change at the evolutionary scale, as shape is inherently multivariate and thus difficult to describe in a bivariate fashion without PCA. We used a pooled sample of adults of 17 species to approximate adult relative skull length (captured on PC1) and used these PC scores in subsequent analyses to correlate different metrics of ontogeny with relative adult skull length. We avoid using a total shape approach in this aspect of the analysis as there are several aspects of skull shape that do not correspond to relative skull length (e.g., foramen position, supraoccipital position) and would thus confound the overall interpretation of our results.

### 2.5 Neurocranial ontogenetic trajectories

Variation in allometric slopes between species was assessed using a size–shape regression and a Procrustes ANOVA to test against the null hypothesis of parallel or homogenous slopes using the “*advanced.procD.lm*” function in *Geomorph*. Where significant interaction terms between log (centroid size) and species were found, additional pairwise *p*‐value comparisons were calculated to determine interspecific differences in allometric slope angles. Allometric trajectories were analyzed for all 17 species and then subdivided between brachycephalic (adult PC1 < 0.00) species and dolichocephalic species (adult PC1 > 0.00) and displayed using a predicted shape vs. log‐centroid size regression. The predicted shape approach of Adams and Nistri (2010) calculates predicted shape values from a regression of shape on size, and plots the first principal component of the predicted values against size in the form of a graphic of the allometric trend.

### 2.6 Measuring ontogenetic modularity/integration

Modularity and integration of shape changes in the neurocranium during ontogeny were evaluated separately in 17 gymnotiform species using 17 landmarks in lateral view. Hypothesized module boundaries were defined as spatially contiguous landmark sets demarking the margins of the braincase (LM 9–17) and face, the latter of which includes the ethmoid and sphenoid regions of the neurocranium (LM 1–8) (Figure 1b). The prebraincase and braincase regions of the actinopterygian skull are defined in Patterson (1975) and Mabee and Trendler (1996) and McCarthy, Sidik, Bertrand, & Eberhart (2016). These two neurocranial regions have been shown to exhibit qualitatively distinct patterns of ontogenetic and phylogenetic shape change in some vertebrates (Emerson & Bramble, 1993), and the gymnotiform clade under investigation (Albert, 2001; Albert, Crampton, Thorsen, & Lovejoy, 2005). The effect of allometry on modularity was also evaluated by taking the residuals of a regression of log‐centroid size vs. shape (Loy, Mariani, Bertelletti, & Tunesi, 1998) for the ontogenetic analysis and analyzing them using the three modularity/integration metrics discussed below.

Recent advances in the theoretical framework of integration and modularity have produced several novel metrics for which to quantify the degree of integration and modularity within and between landmark configurations (Adams, 2016; Bookstein, 2015; Bookstein et al., 2003). We use three metrics to quantify developmental integration, modularity, and disintegration in the face, braincase, and entire neurocranium of 17 gymnotiform species (Figure 1b). Developmental modularity was quantified using the “modularity.test” function in the R package *Geomorph* (Adams & Otárola‐Castillo, 2013). This function quantifies the degree of modularity between hypothesized modules (face and braincase) using a partial least squares analysis (PLS) and compares this to a null distribution of neither integrated or modular structure (Adams, 2016) using the covariance ratio (CR). Significant modularity is found when the CR coefficient is small relative to the null distribution. Lower CR values are interpreted as exhibiting lower covariance between modules (i.e., higher modularity).

Developmental integration was quantified using the “integration.test” function in *Geomorph*. This function quantifies integration using a two‐block PLS analysis (or singular‐warp analysis in the case of this analysis) (Bookstein et al., 2003). The average pairwise PLS correlation functions as the test statistic. Significant integration is determined when this test statistic is larger than the permuted null distribution. The PLS correlation coefficient is interpreted similarly to the (CR) coefficient with higher values corresponding to higher degrees of integration.

To quantify integration across the entire neurocranium in development, the “globalIntegration” function was used in *Geomorph*. This function quantifies global integration using the global integration coefficient (GI) (Bookstein, 2015). In the GI approach, bending energies at various spatial scales are estimated and the log variance of the partial warps is plotted against their corresponding log‐bending energies. The resulting slopes were then used to quantify integration (slopes greater than −1) and disintegration (slopes less than −1). This coefficient was used as a third measure when ambiguous results were returned from the other two metrics (i.e., significant degrees of both integration and modularity in the same species).

### 2.7 Ontogenetic disparity

Ontogenetic disparities of 17 gymnotiform species were calculated using the “moprhol.disparity” function in the R package *Geomorph*. Using this function, ontogenetic disparity is estimated as the Procrustes variance of an ontogenetic series for each species, using residuals of a linear model fit. Procrustes variance is the sum of the diagonal elements of the group sums of squares and cross‐products matrix divided by the number of observations in the group (Zelditch et al., 2012). In our analysis, ontogenetic disparity was calculated after accounting for allometry using centroid size as a continuous covariate in the model. Absolute differences in Procrustes variance were used as test statistics and assessed using permutation, where the vectors of residuals were randomized among groups.

### 2.8 Phylogenetic tree

The hypothesis of phylogeny for Gymnotiformes was based on results from Tagliacollo, Bernt, Craig, Oliveira, & Albert (2016). The phylogeny was built using six genes (5,054 bp) and 223 morphological characters for all gymnotiform species representing 35 extant genera. The full gymnotiform phylogeny of Tagliacollo et al. (2016) was trimmed to the taxon set for which skull morphometric data were available using the drop.tip function in the R package *ape* (Paradis, Claude, & Strimmer, 2004).

### 2.9 Ancestral state estimates

Ancestral states of relative skull length (PC1), global integration, and ontogenetic disparity were calculated in the R package *phytools* using the “contMap” function. This function maps continuous traits on an ultrametric phylogeny and estimates ancestral states at nodes using maximum likelihood and intercalates the states along edges using the second equation in Felsenstein (1985). Traits were mapped onto the pruned Tagliacollo et al. (2016) phylogeny to include only the species in this analysis.

Two separate axes of shape evolution (PC1 and PC2) were visualized using a phylomorphospace analysis. A phylomorphospace diagram depicts the magnitude and direction of shape changes among branches of a clade in a multivariate shape space. This is performed by combining a previously proposed phylogeny with the scatter plots of principal components (PC) scores computed from a PCA and projecting this phylogeny onto a two‐dimensional plane where branch lengths and distances are inferred by the differences in shape between groups using squared‐changed parsimony (Sidlauskas, 2008). Treefiles were built based of the Tagliacollo, Bernt, Craig, Oliveira, & Albert (2015) phylogeny using the software Mesquite v. 2.75 (Maddison & Maddison, 2001) and imported to *MorphoJ* (Klingenberg, 2011) as a Nexus file using the option “map onto phylogeny.”

### 2.10 Phylogenetic signal

Phylogenetic signal in ontogenetic disparity and global developmental integration was measured in the R package *phytools* using Blomberg's *k* (Blomberg, Garland, & Ives, 2003).

### 2.11 Phylogenetic least squares regression

The relationship between ontogenetic covariation and adult relative skull length (PC1) and the relationship between ontogenetic disparity and ontogenetic covariation were tested using a phylogenetic generalized least squares regression (PGLS) to account for phylogenetic nonindependence of traits (Rzhetsky & Nei, 1992). We tested our results using two different models for error structure: Brownian motion and Orenstein‐Uhlenbeck (OU) and used Akaike information criterion (AIC) to evaluate model fit in the R package *nlme* (Pinheiro, Bates, DebRoy, & Sarkar, 2014).

### 2.12 Convergent evolution

A common approach in assessing convergent evolution in continuous trait data is model‐fitting using an OU process (Hansen, 1997; Mahler et al., 2013; Uyeda & Harmon, 2014). In an OU process, continuous traits (*p*) evolve over time (*t*) following the stochastic equation: d*p* (*t*) = α(θ(*t*) − *p*(*t*)) + σd*B* (*t*), where Brownian motion (B(*t*)) evolves toward an inferred trait optima θ. The adaptation rate (α > 0) is used to calculate phylogenetic half‐life (log (2/α)): the time it takes for trait evolution to reach half the distance to θ. If the phylogenetic half‐life is larger than (*t*), then phenotypic evolution converges slowly toward the trait optimum relative to *t*. This results in prolonged variation around the ancestral state, reducing the OU model to a Brownian motion process.

**Figure 2:**
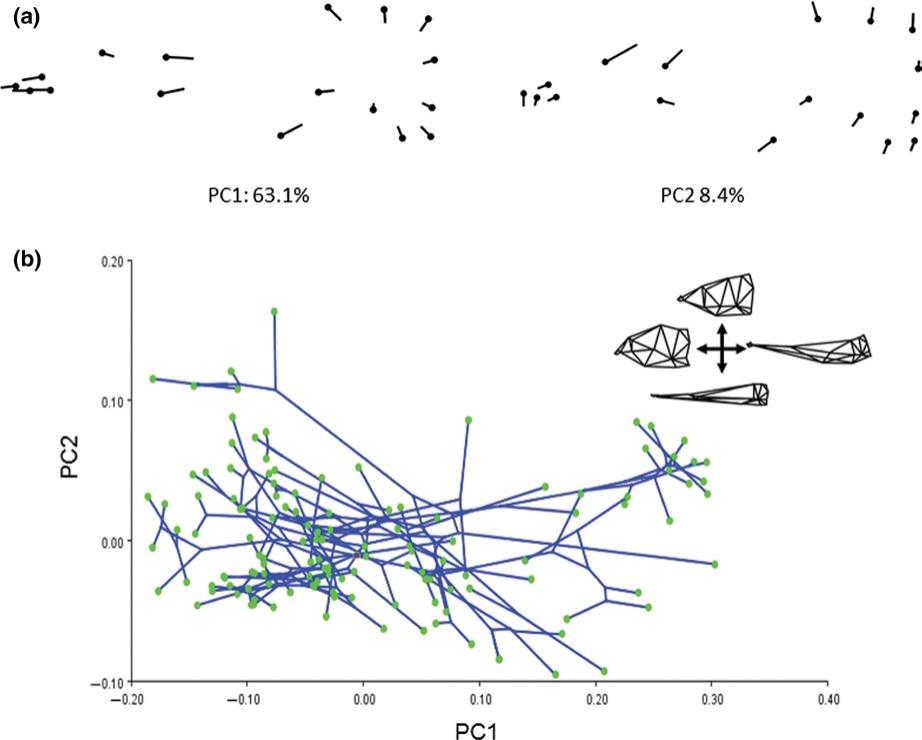
Phylogenetic shape changes in gymnotiform neurocrania for 133 species. (a) Relative warp deformation grids from geometric morphometric analyses showing deformations of the first two principal components (PC). Note the heavy loading of variation in relative skull length (heterocephaly) on PC1. (b) Phylomorphospace analysis depicting the constrained colonization of gymnotiform skull shape. Note the multiple independent colonizations of brachycephalic (low PC1 values) and dolichocephalic skull shape

Convergent evolution of relative skull length (PC1) was evaluated using the *R* package *l1ou* (Khabbazian, Kriebel, Rohe, & Ané, 2016). In this approach, shifts in trait evolution were detected using the lasso method under the OU process (Tibshirani, 1996). Shift magnitudes and positions were evaluated in our analysis using AICc. These shifts were then used to generate a shift configuration. This shift configuration was evaluated for convergence under an OU process also using AICc as the criterion for model selection. Bootstrap supports for shift position and magnitudes were calculated using a nonparametric approach which calculates phylogenetically uncorrelated standardized residuals, one at each node. These residuals are then sampled with replacement and mapped onto the phylogeny to create bootstrap replicates. Bootstraps were replicated 100 times. The use of PCs in macroevolutionary modeling is cautioned against many aspects of macroevolutionary modeling (Revell, 2009; Uyeda et al., 2015). However, no reliable solutions have been determined for modeling PC axes under any process other than Brownian motion. However, Uyeda et al. (2015) found that PCs do not experience appreciable distortion in cases where the leading PC axes explain a large portion of the total variance, which is the case in our dataset (Figure 2). A common finding in macroevolutionary analyses that are biased by the use of PCs is an early‐burst pattern of trait evolution (Harmon et al., 2010; Khabbazian et al., 2016; Uyeda et al., 2015). We tested for this bias by fitting our data to an early‐burst model, a Brownian motion model, and an Orenstein‐Uhlenbeck model in the R package *mvMORPH* (Clavel, Escarguel, & Merceron, 2015). We find that the early‐burst model does not provide the best fit to our data and instead find that the OU model provides the best fit to our data, further suggesting that our use of PCs do not appreciably bias our interpretation of convergent evolution in relative skull length (Table S3).

## 3 Results

### 3.1 Heterocephaly

Here, we refer to variance along the brachycephalic to dolichocephalic axis of craniate skull shape (Retzius & Alexander, 1860) as *heterocephaly,* displayed here as PC1 in Figure 2. Heterocephaly describes a particular pattern of inversely correlated shape changes associated with neurocranial size involving a relative contraction (negative allometry) of the braincase and relative elongation (positive allometry) of the face or snout regions of the skull. By this definition, heterocephaly can refer to neurocranial allometries during growth of a single individual, among individuals of different sizes within a population, among adults of different populations within a species, or among adults between different species, that is, ontogenetic, static, or evolutionary allometries (Voje & Hansen, 2013; Voje et al., 2014). Heterocephalic patterns of variation and diversity have been reported in several other taxa (Tables S4 and S5).

### 3.2 Neurocranial diversity of gymnotiformes

Gymnotiformes display a wide range of craniofacial phenotypes (Figure 2). The first two principal components (PCs) together account for 68.1% of the total variance (Figure 2a,b). Variation along the brachycephalic to dolichocephalic axis (heterocephaly) corresponds to the PC1 axis, with the most brachycephalic skulls possessing the lowest scores (e.g., *Adontosternarchus balaenops*: depicted in inset), and the most dolichocephalic skulls possessing the highest scores (e.g., *Parapteronotus hasemani*). Variation along the axis of skull depth and snout curvature corresponds to the PC2 axis, that is, skulls ranging from a deep to narrow braincase and with a dorsal or ventral inflection of the ethmoid region. *Adontosternarchus balaenops* possesses the deepest skull (highest PC2 score) while *Electrophorus*,* Gymnorhamphichthys,* and *Orthosternarchus* possess the slenderest skulls. Apteronotid species with brachycephalic skulls have a deeper aspect in lateral profile, as compared with apteronotids with elongate snouts or other Gymnotiformes.

### 3.3 Repeated patterns of heterocephalic evolution

**Figure 3:**
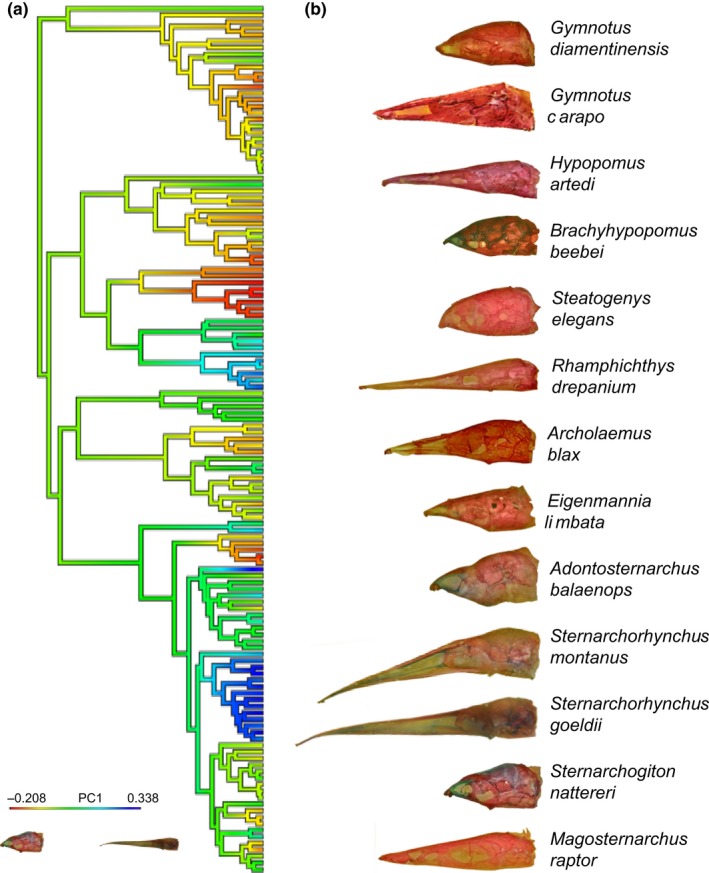
Phylogeny of Gymnotiformes and evolution of heterocephaly. (a) Phylogenetic tree (based on Tagliacollo et al., 2015), trimmed to include 133 species examined for neurocranial morphology. Colored branches indicate heterocephalic variation with blue indicating more dolichocephalic and red more brachycephalic skull shapes (see inset). (b) Neurocrania of gymnotiform species in lateral view illustrating extreme brachycephalic and dolichocephalic morphologies. Note multiple independent evolutionary transitions to dolichocephalic and brachycephalic skull shapes

Among Gymnotiformes, clades with brachycephalic or dolichocephalic skulls, characterized by extreme PC1 values, have evolved multiple times (Figure 3). The apteronotids *Parapteronotus* and *Sternarchorhynchus,* and the rhamphichthyid *Rhamphichthys drepanium,* possess the most dolichocephalic skull shapes with the highest PC1 scores (blue branches in Figure 3a). Species exhibiting the most brachycephalic skull shapes with the lowest PC1 scores include the apteronotids *A. balaenops,* the hypopomid *Brachyhypopomus beebei,* the gymnotid *Gymnotus diamantinensis,* and the rhamphichthyid *Steatogenys elegans* (red branches in Figure 3a).

### 3.4 Neurocranial ontogeny: general patterns

**Figure 4:**
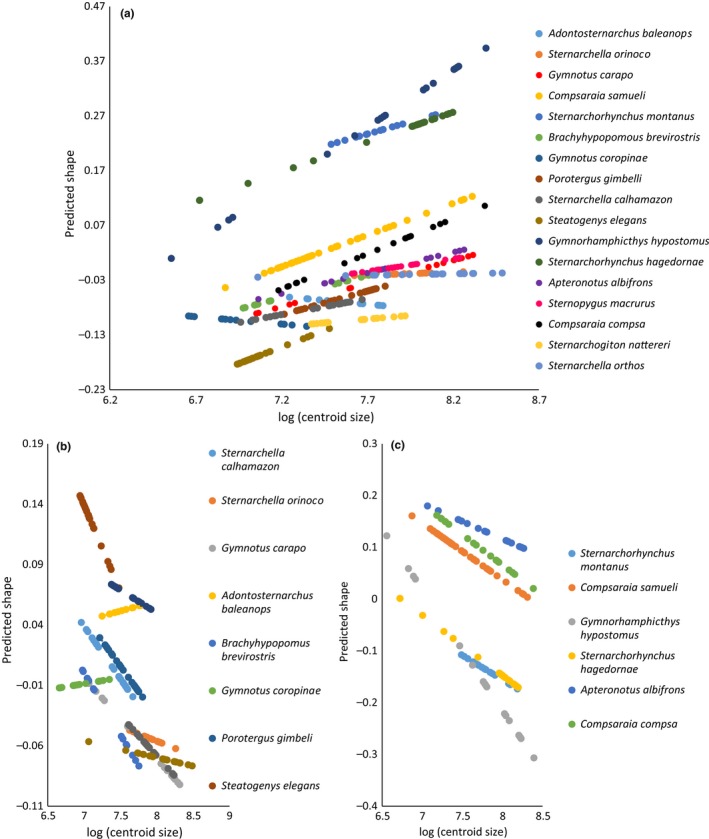
Neurocranial ontogenetic trajectories for predicted shape vs. log‐centroid size. (a) Neurocranial ontogenetic trajectories of 17 gymnotiform species showing the diversity in allometric slopes between species. Procrustes ANOVA for homogeneity of slopes test indicate significant (*p* = 0.001) differences between allometric slope angles. (b) Ontogenetic trajectories of 10 brachycephalic (PC1 < 0.00) species showing the diversity of allometric slopes between species with similar degrees of heterocephaly. Procrustes ANOVA for homogeneity of slopes test indicate significant (*p* = 0.001) differences between allometric slopes. (c) Ontogenetic trajectories of six dolichocephalic (PC1 > 0.00) species showing more similarity in slopes between species. Procrustes ANOVA for homogeneity of slopes test indicate significant (*p* = 0.001) differences between allometric slopes within this group of species

The ontogenies that construct the craniofacial phenotypes of Gymnotiformes are highly variable in slope between species (Table 2) (Figure 4). In the full‐species ontogenetic analysis, size explains 19% of the shape variation while species identity explains 67%. The Procrustes ANOVA recovered significant interaction terms (*p* = 0.02) between size and species indicating that slopes differed significantly between species. Heterocephaly corresponded to the first principal axis among a pooled sample of adult specimens for each species (Figure S1). Ontogenies were subsequently divided into brachycephalic (adult PC1 < 0.00) and dolichocephalic (adult PC1 > 0.00) classes for further statistical evaluation.

**Table 2:** Procrustes ANOVA of ontogentic slope angles for 17 species of gymnotiform fishes. Bold values indicate significance

Full ontogenetic ANOVA	*df*	SS	MS	Rsq	*F*	*Z*	Pr(>*F*)
Log(size)	1	1.595	1.595	0.193	586.043	20.423	**.002**
Species	16	5.494	0.343	0.665	126.127	14.061	**.002**
Log(size):species	16	0.282	0.018	0.034	6.472	5.262	**.002**
Residuals	327	0.89	0.003				
Total	360	8.262					

Three tiers of ontogenetic disparity (ontogenetic Procrustes variance) were found in analysis of pairwise comparisons (Tables 3 and S6). Dolichocephalic species with tube‐shaped snouts (*Sternarchorhynchus* and *Gymnorhamphichthys*) differed significantly from all other non‐tube‐snouted species in ontogenetic disparity, with these taxa exhibiting the highest ontogenetic disparities. Additional species‐specific differences were also found between *Gymnorhamphichthys* and *Sternarchorhynchus*. Dolichocephalic species without a tube‐shaped snout (*Compsaraia* and *Apteronotus*) differed significantly from the most brachycephalic species (*Adontosternarchus*,* Sternarchogiton*,* Steatogenys,* and *Sternarchella orthos*). Most brachycephalic species did not differ significantly in ontogenetic disparity (except *P. gimbeli*).

**Table 3:** Ontogenetic disparities and average relative skull lengths of adult specimens (PC1) of 17 gymnotiform species

Species	Ontogenetic disparity	PC1
*A. albifrons*	0.008	0.000
*A. baleanops*	0.020	−0.087
*B. brevirostris*	0.010	−0.053
*C. compsa*	0.006	0.039
*C. samueli*	0.006	0.097
*G. hypostomus*	0.070	0.305
*G. carapo*	0.014	−0.021
*G. coropinae*	0.014	−0.144
*P. gimbelli*	0.006	−0.079
*S. calhamazon*	0.011	−0.096
*S. elegans*	0.016	−0.164
*S. hagedornae*	0.048	0.223
*S. macrurus*	0.008	−0.035
*S. montanus*	0.052	0.234
*S. nattereri*	0.018	−0.123
*S. orinoco*	0.011	−0.048
*S. orthos*	0.018	−0.049

### 3.5 Neurocranial ontogeny: brachycephalic patterns

Brachycephalic species exhibit a diverse range of ontogenetic slope angles (Figure 4). A Procrustes ANOVA recovered pairwise species‐specific differences in slope angles (Table 4). The slope angles appear to cluster in two main groups, the first of which is comprised of species with shallow slope angles (≤90°) (*A. balaenops*,* G. coropinae*,* S. orthos,* and *Sternarchella orinoco*). The second group is comprised of species with slope angles larger than 90° (*S. calhamazon, P. gimbeli, S. elegans, S. macrurus,* and *B. brevirostris*). Here, even closely related brachycephalic species (i.e., in the same genus: *S. calhamazon* vs. *S. *orthos, *G. carapo* vs. *G. coropinae*) differ significantly in ontogenetic slope angle.

**Table 4:** Pairwise comparisons of ontogenetic allometric slope angles for 11 brachycephalic species of gymnotiform fishes (PC1 < 0.00)

Brachycephalic ANOVA	*df*	SSE	SS	*R* ^2^	*F*	*Z*	Pr(>*F*)
Log(centroid size)	227	2.045					
Log(centroid size) + species	217	0.613	1.431	.549	50.679	14.05	**.001**

Pairwise comparisons of slope angle (degrees)											
Species	*S. calhamazon*	*S. nattereri*	*S. orthos*	*S. orinoco*	*G. carapo*	*A. baleanops*	*B. brevirostris*	*G. coropinae*	*P. gimbeli*	*S. elegans*	*S. macrurus*
*S. calhamazon*	1	0.197	**0.01**	0.071	**0.048**	**0.029**	0.819	**0.023**	0.481	0.681	0.365
*S. nattereri*	0.197	1	0.121	0.069	**0.042**	0.138	0.322	**0.045**	0.285	0.274	0.343
*S. orthos*	**0.01**	0.121	1	**0.019**	**0.001**	0.255	**0.003**	**0.033**	**0.034**	0.17	0.052
*S. orinoco*	0.071	0.069	**0.019**	1	0.058	0.183	0.051	0.368	0.19	**0.048**	0.273
*G. carapo*	**0.048**	**0.042**	**0.001**	0.058	1	0.058	0.125	**0.033**	0.296	0.182	0.271
*A. baleanops*	**0.029**	0.138	0.255	0.183	0.058	1	**0.049**	0.354	**0.045**	0.062	0.369
*B. brevirostris*	0.819	0.322	**0.003**	0.051	0.125	**0.049**	1	**0.01**	0.601	0.533	0.523
*G. coropinae*	**0.023**	**0.045**	**0.033**	0.368	**0.033**	0.354	**0.01**	1	**0.01**	**0.013**	0.178
*P. gimbeli*	0.481	0.285	**0.034**	0.19	0.296	**0.045**	0.601	**0.01**	1	0.733	0.381
*S. elegans*	0.681	0.274	0.17	**0.048**	0.182	0.062	0.533	**0.013**	0.733	1	0.163
*S. macrurus*	0.365	0.343	0.052	0.273	0.271	0.369	0.523	0.178	0.381	0.163	1

### 3.6 Neurocranial ontogeny: dolichocephalic patterns

Dolichocephalic species appear to exhibit less diversity in slope angles, with all observed angles larger than 90° (Figure 4). However, a Procrustes ANOVA found significant differences in slope angle between some dolichocephalic species ontogenies (Table 5). *Gymnorhamphichthys hypostomus* possesses the largest slope angle and was found to differ significantly from all other dolichocephalic species (excluding *S. montanus*) in pairwise comparisons of slopes angles. Among dolichocephalic species, closely related taxa (i.e., in the same genus) were found to possess statistically indistinguishable slope angles (except *C. samueli*).

**Table 5:** Pairwise comparisons of ontogenetic allometric slope angles for six dolichocephalic species of gymnotiform fishes (PC1 < 0.00)

	*df*	SSE	SS	*R* ^2^	*F*	*Z*	Pr(>*F*)
Log(centroid‐size)	130	2.375					
Log(centroid‐size) + species	125	0.516	1.859	.598	90.066	16.026	**.001**

	Pairwise comparisons of slope angle (degrees)					
Species	*S. montanus*	*C. samueli*	*G. hypostomus*	*S. hagedornae*	*A. albifrons*	*C. compsa*
*S. montanus*	1	0.497	0.344	0.417	0.339	0.346
*C. samueli*	0.497	1	**0.007**	**0.045**	0.214	0.213
*G. hypostomus*	0.344	**0.007**	1	**0.022**	**0.049**	**0.041**
*S. hagedornae*	0.417	**0.045**	**0.022**	1	0.112	0.103
*A. albifrons*	0.339	0.214	**0.049**	0.112	1	0.209
*C. compsa*	0.346	0.213	**0.041**	0.103	0.209	1

### 3.7 Ontogenetic modularity and integration

The ontogenetic series of skulls in 17 gymnotiform species were evaluated for covariation between two hypothesized developmental modules: the braincase and face regions (Figure 1b) using the CR coefficient and the PLS correlation coefficient. Global integration of the entire landmark structure was also evaluated using the GI coefficient (Table 6). Across all three metrics, a significant relationship was recovered using a PGLS regression between ontogenetic integration (*p* ≤ 0.001), ontogenetic modularity (*p* = 0.04), global developmental integration (*p* ≤ 0.001), and adult relative skull length for each species approximated using average PC1 scores of adult specimens (Figure S2 and Table 7). In these analyses, Brownian motion was found to be the model of best fit to each regression when compared to an OU model. The third‐most brachycephalic species (*Adontosternarchus*) displayed significant patterns of ontogenetic modularity and possessed lower ontogenetic CR coefficients while also exhibiting a lower ontogenetic GI coefficient. As expected, this same species failed the test of integration quantified by the PLS correlation coefficient. The inverse was true for the most dolichocephalic species (PC1 > 0) (*Apteronotus, Compsaraia, Sternarchorhynchus,* and *Gymnorhamphichthys*) that exhibit significant patterns of ontogenetic integration but failed the test of modularity and exhibited the highest ontogenetic GI coefficients. Interestingly, some species exhibited significant patterns of both ontogenetic integration and modularity (i.e., *S. orinoco, S. calhamazon, Gymnotus*, and *Brachyhypopomus*) and these species had intermediate GI values ranging from −0.38 to −0.54. The most brachycephalic species (*S. elegans*) exhibits a significant pattern on developmental integration which was not expected given its brachycephalic skull shape and low ontogenetic GI value. The second‐most brachycephalic species (*Sternarchogiton nattereri*) was found to be neither significantly integrated nor modular in ontogeny. However, this species returned the lowest ontogenetic GI value of any of the sampled species.

**Table 6:** Ontogenetic modularity and integration of the neurocranium for 17 species of gymnotiform fishes. Bold values indicate statistical significance

Species	Ontogenetic GI	Ontogenetic CR	*p*‐Value	r.pls	*p*‐Value
*A. balaenops*	−0.52	0.883	**.034**	0.791	.088
*A. albifrons*	−0.54	1.029	.274	0.901	**.003**
*B. brevirostris*	−0.54	0.876	**.004**	0.873	**.001**
*C. compsa*	−0.58	1.083	.676	0.951	**.001**
*C. samueli*	−0.63	0.999	.092	0.915	**.001**
*G. hypostomus*	−1.01	1.098	.21	0.966	**.001**
*G. carapo*	−0.49	0.992	**.024**	0.914	**.001**
*G. coropinae*	−0.38	0.85	**.004**	0.734	**.001**
*P. gimbeli*	−0.52	0.979	.228	0.754	**.045**
*S. elegans*	−0.34	1.006	.432	0.919	**.001**
*S. calhamazon*	−0.50	0.818	**.008**	0.816	**.001**
*S. orinoco*	−0.38	0.921	**.006**	0.822	**.041**
*S. orthos*	−0.33	0.924	.114	0.703	**.015**
*S. nattereri*	−0.03	1.135	.902	0.994	.844
*S. hagedornae*	−0.74	1.048	.272	0.977	**.001**
*S. montanus*	−0.75	1.069	.352	0.988	**.001**
*S. macrurus*	−0.52	0.822	**.04**	0.622	.275

**Table 7:** AIC values for PGLS models (Brownian motion & OU) of three metrics of ontogenetic integration and modularity and adult relative skull length. Bold indicates statistical significance

	Brownian motion	OU	Bp	Oup
GI	−40.59	−34.05	**>0.00**	**>0.00**
CR	−18.6	−12.67	**0.01**	0.95
r.pls	1.17	−12.21	**0.04**	0.53

Allometric correction had no significant effect on ontogenetic PLS correlation coefficients (*p* = 0.11) or global integration coefficients (*p* = 0.469). However, allometric correction did significantly affect CR coefficient values (*p* = 0.026) (Table S7). Despite the lack of significant differences, all of the dolichocephalic species experienced slight to large decreases in covariation coefficient values after allometric correction.

A significant relationship was recovered between ontogenetic disparity, ontogenetic global integration, and the ontogenetic modularity (Table 8). Significant pairwise differences were also found between ontogenetic disparities between species with the most dolichocephalic skulls (*Sternarchorhynchus* and *Gymnorhamphichthy*s) differing from all of the more brachycephalic species (Table 3). No significant relationship was found between integration (PLS coefficient) and ontogenetic disparity (*p* = 0.11). Using the GI coefficient, it was also found that more integrated species exhibited higher levels of ontogenetic disparity (Figure S3). Brownian motion was found to be the model of best fit for all metrics of modularity/integration and ontogenetic disparity when compared to an OU model (Tables 6 and 7).

**Table 8:** AIC values for PGLS models of three metrics of ontogenetic integration/modularity and ontogenetic disparity. Bold indicates statistical significance

	Brownian motion	OU	Bp	Oup
GI	−78.27	−89.14	**>0.00**	**0.005**
CR	−58.28	−80.96	**>0.00**	0.413
r.pls	−41.26	−80.25	0.11	0.8

### 3.8 Evolution of developmental integration and ontogenetic disparity

**Figure 5:**
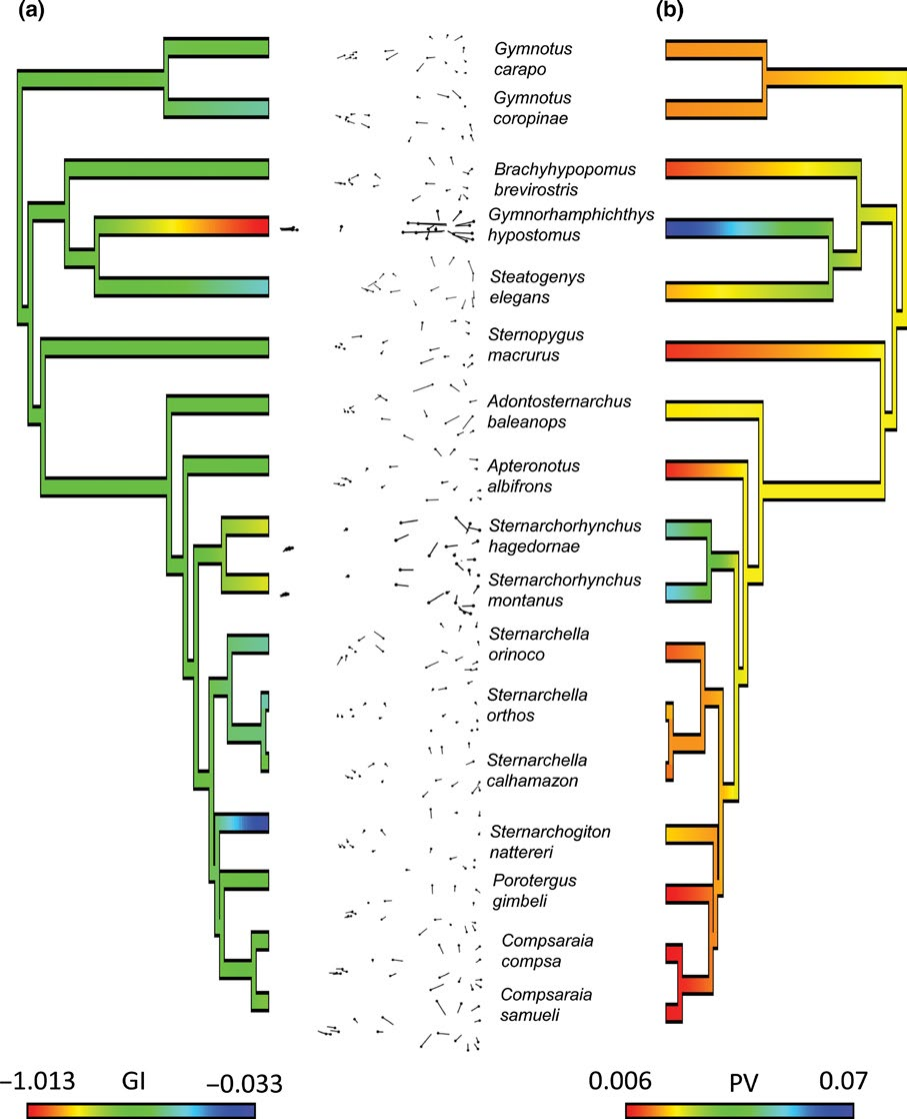
Phylogenetic changes in neurocranial ontogeny of 17 species of Gymnotiformes plotted on trimmed phylogeny from Figure 4. (a) Continuous trait evolution of ontogenetic neurocranial integration (GI coefficients); lower values (blue) indicate disintegrated development of the neurocranium. Insets depict ontogenetic shape deformation for each species. (Note) common pattern of heterocephaly that can be observed in most of the ontogenetic warps. (b) Continuous trait evolution of ontogenetic disparity (Procrustes Variance); lower values (red) indicate lower ontogenetic disparity. Note the plesiomorphic condition in Gymnotiformes is to have intermediate levels of neurocranial integration with a GI value of about −0.50 and that species with extreme (high and low) GI values have evolved several times each. Note also that the most dolichocephalic species (*Sternarchorhynchus* and *Gymnorhamphichthys*) also exhibit the highest ontogenetic disparity

The ancestral state of neurocranial global integration (GI) Gymnotiformes is intermediate level of integration (GI = −0.50) (Figure 5a). Here, the independent evolution of extreme GI values is observed in *G. coropinae*,* S. calhamazon*,* S. orinoco*,* S. elegans*,* S. nattereri,* and *Sternarchorhynchus*. No significant phylogenetic signal was recovered for global integration (*p* = 0.74).

Ancestral states of ontogenetic disparities were estimated to be slightly higher than intermediate levels (PV = 0.03; Figure 5b). Species that displayed intermediate levels of integration (i.e., *Apteronotus*,* Compsaraia,* and *Sternopygus*) displayed the least ontogenetic disparity followed by other species with lower levels of ontogenetic integration. Conversely, highly integrated species exhibited the largest ontogenetic disparities. Both these patterns are estimated to have evolved multiple times independently, and no significant phylogenetic signal is observed in the ontogenetic disparities of these species (*p* = 0.97).

### 3.9 Convergent evolution in heterocephaly

**Figure 6:**
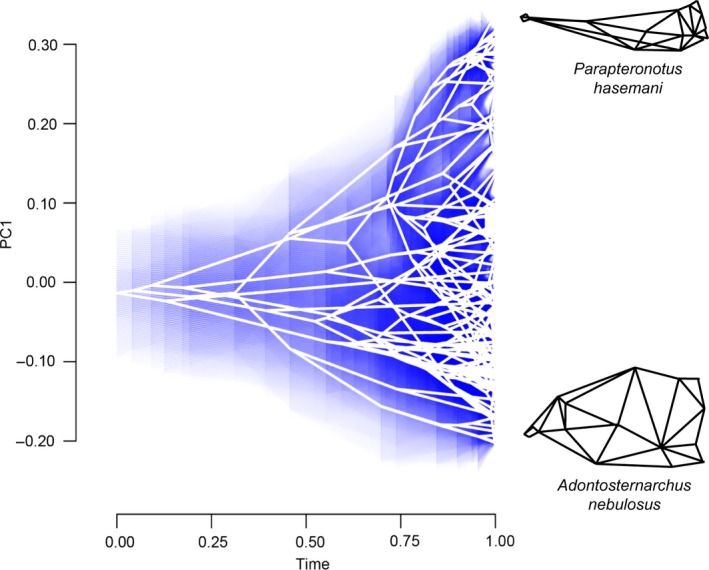
Phenogram of heterocephalic evolution for 133 gymnotiform species plotted against time. Phylogeny based on Tagliacollo et al. (2015). Note multiple independent colonizations of both low (brachycephalic) and high (dolichocephalic) PC1 scores. Blue shading indicates 95% confidence limits. Wireframe drawings illustrate skulls with extreme neurocranial shapes

**Figure 7:**
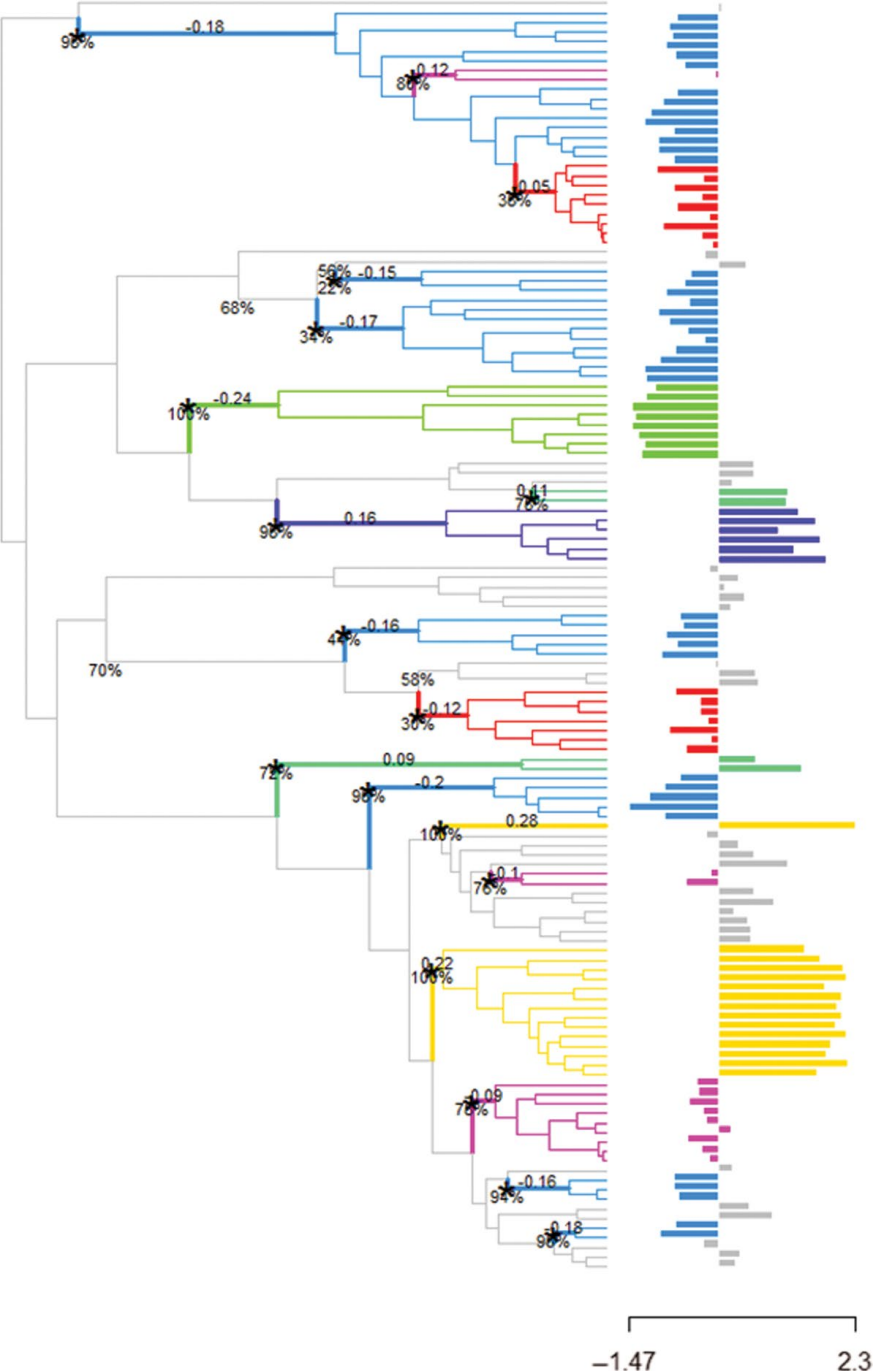
Convergent evolution of heterocephaly in 133 species of Gymnotiformes. Shift magnitudes and bootstrap support values are plotted at nodes. Histogram trait values on the left represent brachycephalic phenotypes, and trait values on the right represent dolichocephalic phenotypes.

Brachycephalic and dolichocephalic skulls evolved 18 independent times within Gymnotiformes (Figure 6). Of these 18 shifts in skull shape, 16 were shifts to convergent phenotypes (Figure 7). Three brachycephalic convergent regimes were estimated across the phylogeny, two of which had bootstrap support values over 70% for most shifts (purple and light blue), with the light blue regime being the largest. This regime corresponded to the most extreme brachycephalic phenotypes and evolved at least once in four of the five gymnotiform families. This regime is characterized by species with highly foreshortened and gracile snouts with reduced or completely absent dentition in the oral jaws (except *Gymnotus*). Additionally, this regime includes two clades of specialized river‐channel planktivores (*Adontosternarchus* and *Rhabdolichops*) (Marrero & Winemiller, 1993). The light blue regime is also comprised of several species whose convergence in brachycephalic skull shape along with other similar craniofacial characters resulted in taxonomic confusion in the placement of these species in the phylogenetic classification in previous analyses (i.e., *Adontosternarchus*,* Sternarchogiton,* and *Porotergus*) (Albert, 2001). Another brachycephalic regime (purple) included slightly less brachycephalic species (Sternarchellini, *Gymnotus,* and *Apteronotus*), and many of these species possess robust dentition and have been identified as trophic generalists feeding on a wide range of prey items ranging from macroinvertebrates to small fishes; two species within this clade are known to be specialized piscivores that feed exclusively on the scales and tales of other electric fishes suggesting that this phenotype is highly adaptable (Ivanyisky & Albert, 2014; Lundberg, Fernandes, Albert, & Garcia, 1996; Marrero & Winemiller, 1993). The red regime was found to have little bootstrap support in this analysis. The dark green brachycephalic regime is occupied by the Steatogenae and returned the second highest shift magnitude do to its close relationship with the highly dolichocephalic Rhamphichthyinae. This regime is not convergent, but represents a unique highly brachycephalic phenotype. Steatogenae includes electric fishes with the smallest body sizes (*Hypopygus*) and planktivorous species (*S. elegans*) (Marrero & Winemiller, 1993; Winemiller & Adite, 1997).

Two convergent dolichocephalic regimes were estimated in the analysis (light green and yellow). Interestingly, our results find no support for convergence of the tube‐snouted clades of *Rhamphichthys* and *Sternarchorhynchus*; instead, *Rhamphichthys* is a nonconvergent regime, and *Sternarchorhynchus* is convergent with *Parapteronotus hasemani* (yellow). Two other tube‐snouted clades (Sternarchorhamphinae and *Gymnorhamphichthys*) constitute their own convergent regime (light green).

## 4 Discussion

Here, we present evidence for a developmental pattern inferred to bias the production of skull shape toward brachycephalic adult phenotypes. We find that the disintegration and sometimes modularization of the neurocranium during development is strongly linked to the production of brachycephalic adult phenotypes. Additionally, we find that strong patterns of developmental integration are linked to more dolichocephalic phenotypes. We also find that species that exhibit developmental disintegration and modularity generally exhibit less ontogenetic disparity while more integrated species were found to exhibit more ontogenetic disparity. Despite significant differences in ontogenetic disparity between brachycephalic and dolichocephalic species, brachycephalic species were not found to differ significantly from each other in most instances. However, more species‐specific differences in ontogenetic slope angle were found between closely related brachycephalic than dolichocephalic taxa among congeners. This suggests that while ontogenetic disparities are fairly constant among brachycephalic species, differences in slope angles may produce shape diversity within similar ranges of ontogenetic disparity.

We estimate that the ancestral heterocephalic condition within Gymnotiformes was a skull of intermediate relative length, similar in proportions to the extant species *Apteronotus albifrons* and *Sternopygus macrurus*. This result is consistent with earlier published estimates of the ancestral gymnotiform skull shape (Albert & Fink, 2007; Albert et al., 2005; Gregory, 1933). We also estimate ancestral states of developmental integration and find intermediate values consistent with the degree of developmental integration in *S. macrurus*. We estimate the ancestral state of ontogenetic disparity to be slightly lower than the median of measured tip values. We find no significant phylogenetic signal in developmental integration or ontogenetic disparity, suggesting that these patterns are highly plastic, allowing them to evolve multiple times independently.

### 4.1 Developmental biases in the production of brachycephalic skulls

Recent advances in the field of evo‐devo have elucidated underlying developmental mechanisms that may modulate continuous variation in facial region during development along the heterocephalic axis (Hu & Marcucio, 2009a, 2009b; Hu et al., 2003, 2015; Marcucio et al., 2005; Parsons, Taylor, Powder, & Albertson, 2014). One such mechanism is the modulation of a gradient of *Shh* and *Fgf8* signaling molecules from the forebrain which can result in more or less brachycephalic phenotypes (Hu & Marcucio, 2009a; Hu et al., 2003; Marcucio et al., 2005). These signaling molecules act as an integrating force across the neurocranium between face and braincase regions. Perturbations to this signaling gradient that result in the collapse of the facial primordia are expected to leave a less integrated signal within the neurocranium. Our findings support this hypothesis, as most brachycephalic species exhibit more disintegrated ontogenies than do dolichocephalic species. However, only in certain cases of brachycephaly were significant degrees of modularity recovered between the face and braincase (Table 5). This finding suggests that while ontogenetic disintegration of the neurocranium may coincide with brachycephalization, this disintegration does not guarantee significant modularization of the neurocranium.

It is possible that other signaling processes may work in conjunction to further influence brachycephalization. Parsons et al. (2014) found that expanded *Wnt/*β*‐*catenin signaling during craniofacial development worked to lock in larval craniofacial phenotypes through accelerated rates of bone deposition. The expansion of the signaling was found to produce a brachycephalic skull with a convex dorsal surface. This craniofacial phenotype resembles the adult phenotype of *S. elegans* where the skull is highly brachycephalic with a convex dorsal margin and well ossified (Figure 3). This species was also found to be highly integrated in development despite being brachycephalic. This unusual developmental patterning may be the result of additional signaling molecular pathways that can further alter a brachycephalic skull.

All the signaling molecules discussed above are known to perform multiple functions during development (Dworkin, Boglev, Owens, & Goldie, 2016; Harada, Sato, & Nakamura, 2016; McCarthy et al., 2016; Sudheer et al., 2016; Wada et al., 2005). It is therefore unlikely that modulation of these signaling molecules is regulated by a single gene. Instead, it is more likely that this signaling and reception are governed by a large pleiotropic gene regulatory network. In this scenario, a mutation anywhere in the network could perturb the signaling from the forebrain, or the reception of the signal in the facial primordia, ultimately producing a brachycephalic face as a plastic response. In other words, it may be easier to break the integration of the face and braincase modules to produce a brachycephalic phenotype than to become more integrated and grow a longer face. Such a developmental bias is predicted to result in more instances of evolutionary convergence toward brachycephalic than dolichocephalic phenotypes.

### 4.2 Convergent evolution under heterocephaly

Biases in the production of one phenotype over another are expected to result in more widespread convergence of the favored than the less favored phenotypes (Smith et al., 1985). Here, we estimate three convergent brachycephalic phenotypes and find widespread convergence of brachycephalic skulls across four of the five major gymnotiform clades (Figure 7). In contrast, we recover only two convergent dolichocephalic regimes, both of which are confined to two major gymnotiform clades (Apteronotidae and Rhamphichthyidae). In general, the dolichocephalic regimes correspond to tube‐snouted faces (except *Parapteronotus*). Across Gymnotiformes, tube snouts have evolved four times (Albert, 2001). However, only once in this analysis are they found to be convergent (light green, Sternarchorhamphinae and *Gymnorhamphichthys*). These phenotypes are characterized by short gapes and nares positioned at the anterior end of the snout. Similar tube‐snouted phenotypes evolved separately in other teleost groups and have been associated with a specialized form of grasp‐suction feeding (Bergert & Wainwright, 1997; Marrero & Winemiller, 1993; Ward & Mehta, 2010; Winemiller & Adite, 1997). It is therefore likely that selective and functional constraints associated with the feeding mechanics of tube suction feeding have contributed to convergent evolution of this phenotype.

These limited structural and functional similarities observed among independently evolved dolichocephalic gymnotiforms stand in strong contrast to the substantial structural and functional diversity observed in brachycephalic taxa. An example can be found in the light blue regime (*Adontosternarchus, Gymnotus,* and *Sternarchogiton*) where despite all species possessing gracile rounded and foreshortened skulls, certain clades have evolved robust oral dentition (*Gymnotus*) associated with piscivory while other clades have lost oral dentition all together and exhibit planktivorous habits (*Adontosternarchus*). Two clades in this regime (*Adontosternarchus* and *Sternarchogiton*) were found to also undergo limited degrees of ossification during growth of the facial region, thus retaining a juvenilized appearance as compared with a more heavily ossified *Magosternarchus* skull. A similar pattern is observed in the purple regime characterized by *Sternarchella* and other *Gymnotus* taxa*,* which exhibit a diverse array of oral dentitions and trophic ecologies while also possessing similarly foreshortened faces (Albert et al., 2005; Ivanyisky & Albert, 2014). These differences in morphologies and ecologies associated with foreshortened faces suggest that the brachycephalic phenotype is highly adaptable to a wide range of ecologies and functions whereas dolichocephalic skulls are potentially more narrowly adapted in this clade.

In this study, we evaluate the hypothesis of a developmental bias toward the production of brachycephalic phenotypes in gymnotiform electric fishes. We find that foreshortened brachycephalic skulls exhibit disintegrated patterns of craniofacial development, while elongate dolichocephalic species exhibit more integrated patterns of development. We also find a relationship between disintegration and ontogenetic disparity, in which species with a more integrated ontogeny exhibit larger ontogenetic disparities. We also report several convergent regimes within the brachycephalic phenotypes, with a wide phylogenetic distribution, as compared to the fewer or more restricted phylogenetic distribution of dolichocephalic skull shapes. Our data support the hypothesis that underlying signaling pathways during development bias phenotypic production toward brachycephalic skull shapes, thus leading to widespread convergence of this trait within Gymnotiformes. This developmental bias may be present in other vertebrate clades, as heterocephalic variation is widespread across many vertebrate taxa.

## Conflict of Interest

None declared.
