# Supplementary figures and images for: Why the short face? Developmental disintegration of the neurocranium drives convergent evolution in neotropical electric fishes
Source: Ecol Evol. 2017 Feb 15;7(6):1783–801. doi: 10.1002/ece3.2704 (PMC5355199; doi:10.1002/ece3.2704)

Figure S1

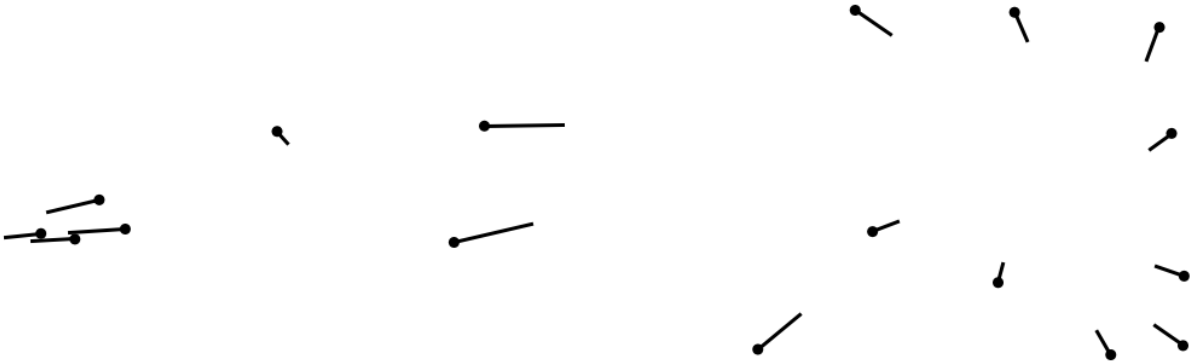

PC1

Supplement: Supplementary file 1 [file ECE3-7-1783-s001.pdf]

Figure S2

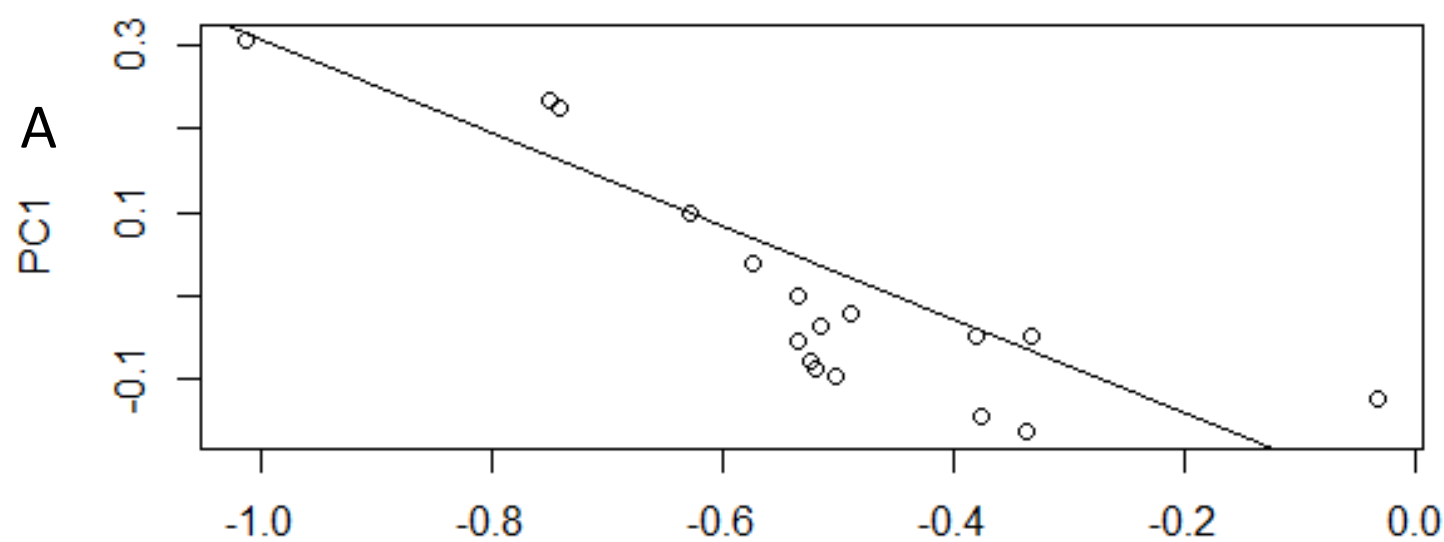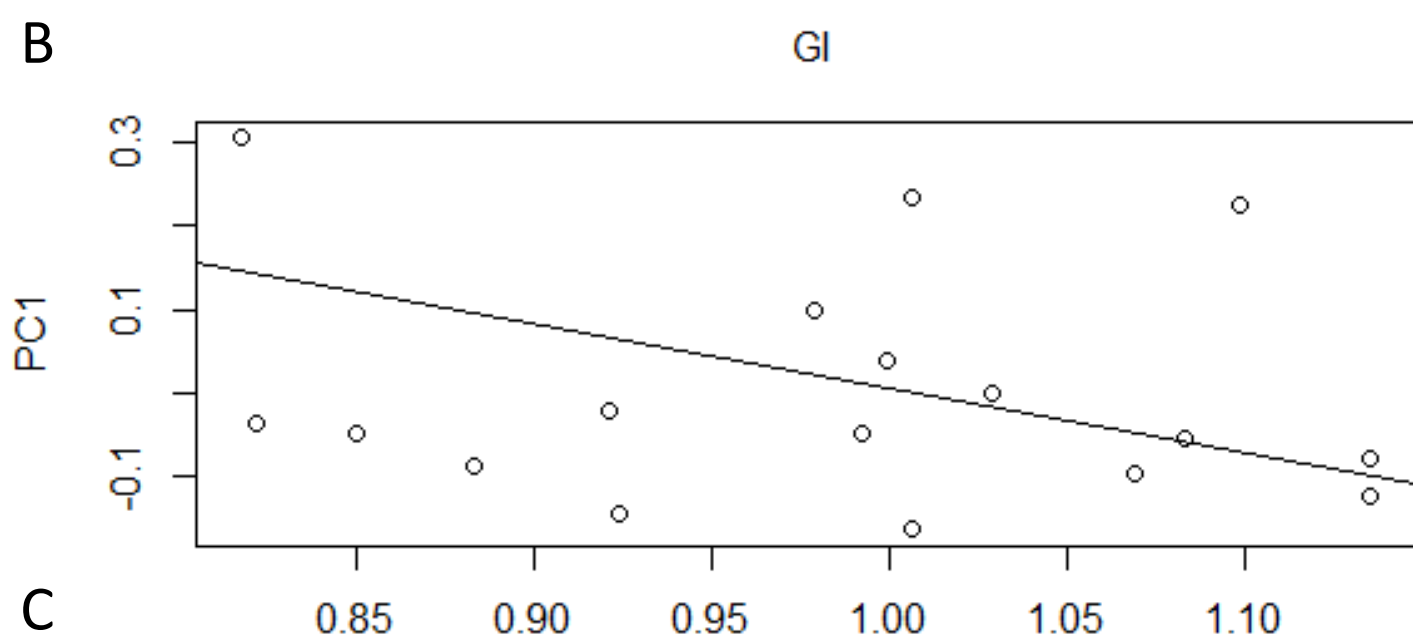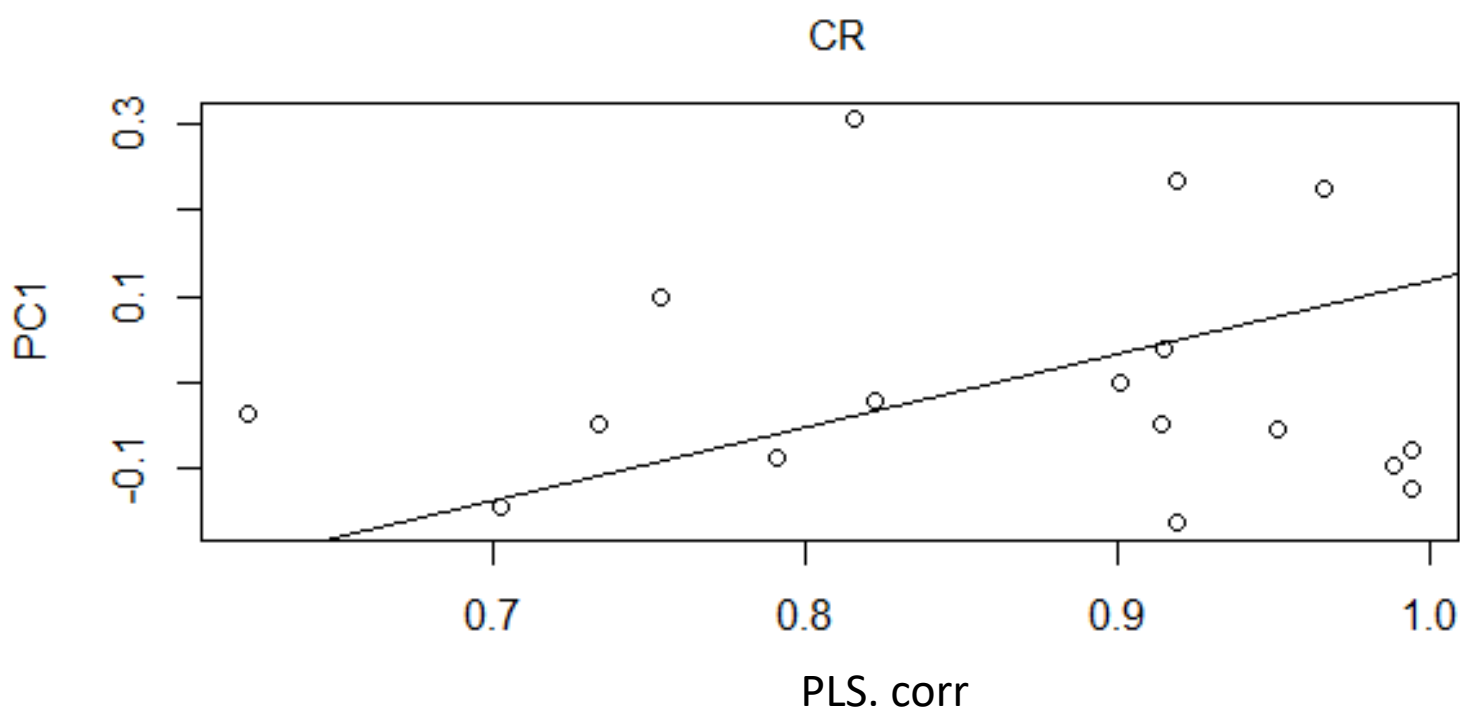

Supplement: Supplementary file 2 [file ECE3-7-1783-s002.pdf]

Figure S3

A

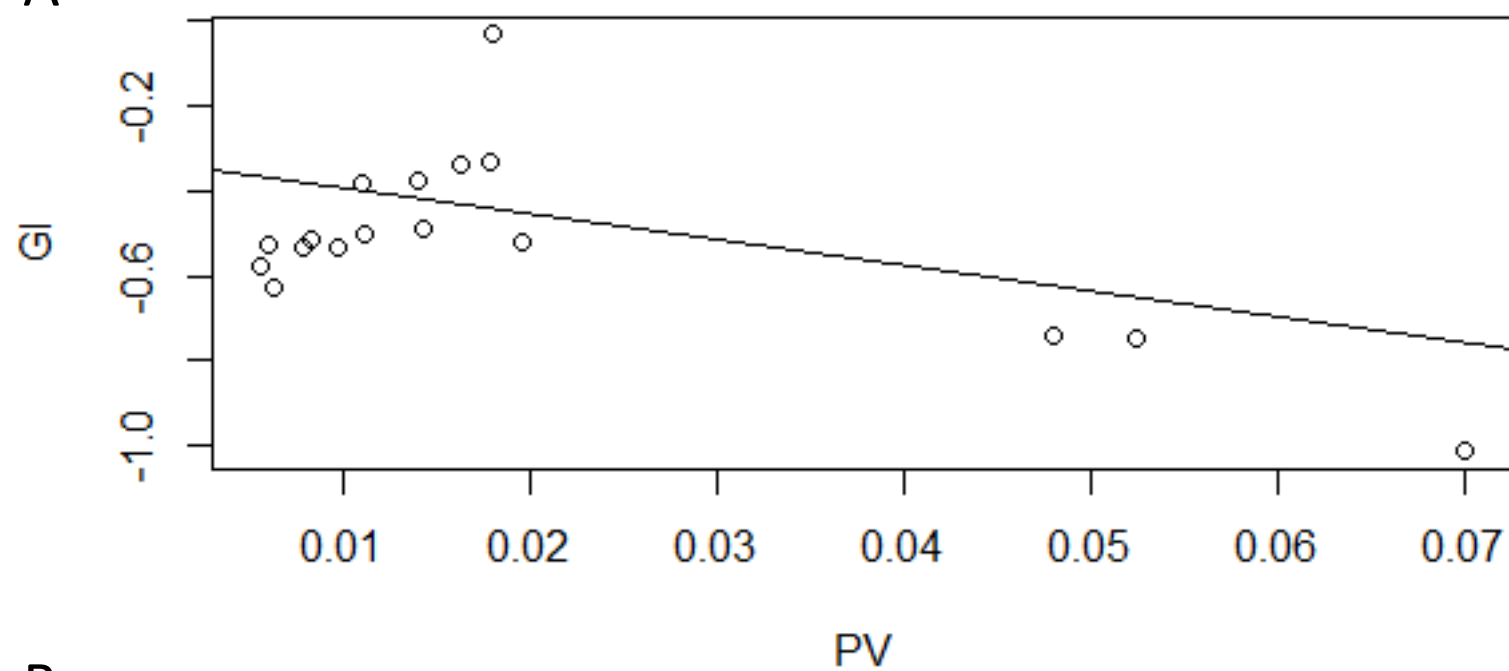

B

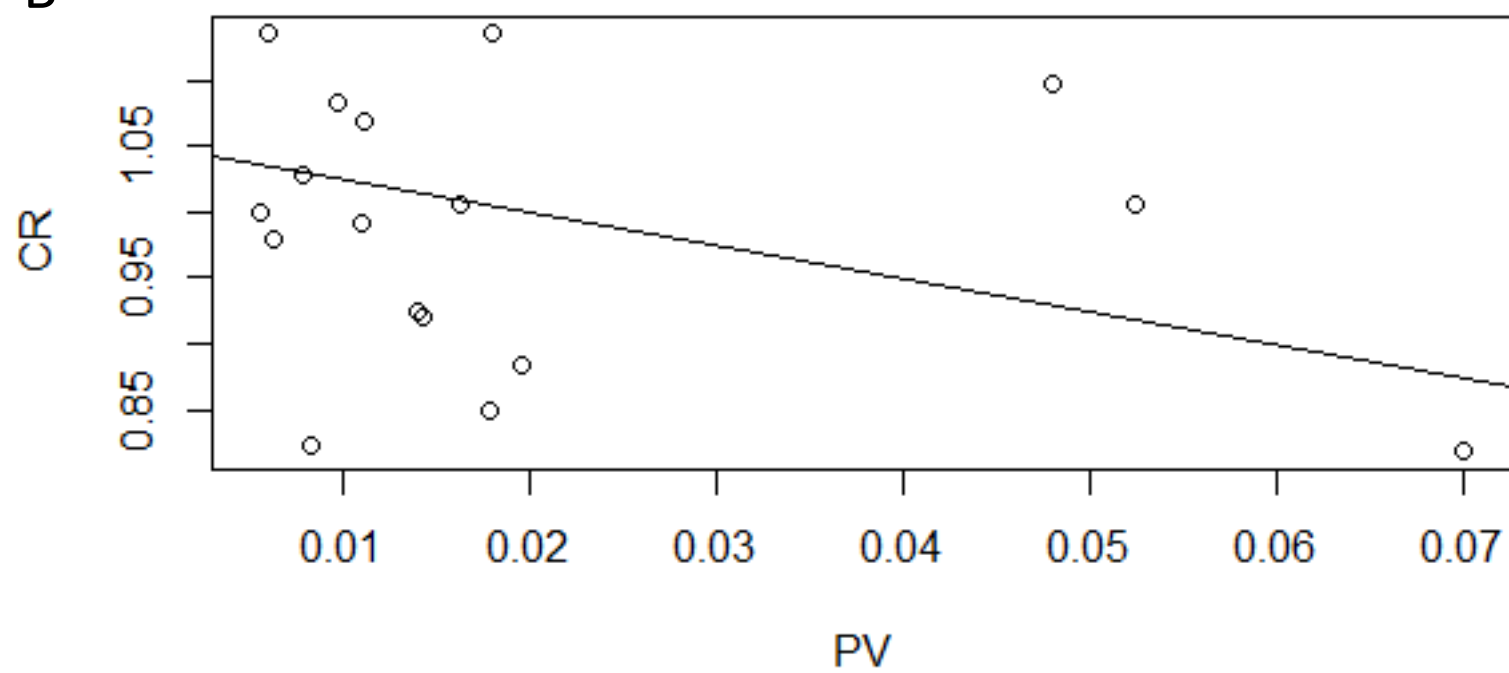

Supplement: Supplementary file 3 [file ECE3-7-1783-s003.pdf]
